# Insights into PSII’s S_3_Y_Z_^•^ State: An Electronic and Magnetic Analysis

**DOI:** 10.1021/acs.jpclett.3c03026

**Published:** 2024-01-08

**Authors:** Felix Rummel, Thomas Malcomson, Maxim Barchenko, Patrick J. O’Malley

**Affiliations:** † Department of Chemistry, School of Natural Sciences, The University of Manchester, Manchester M13 9PL, United Kingdom; ‡ School of Biosciences, Cardiff University, Museum Avenue, Cardiff CF10 3AX, United Kingdom

## Abstract

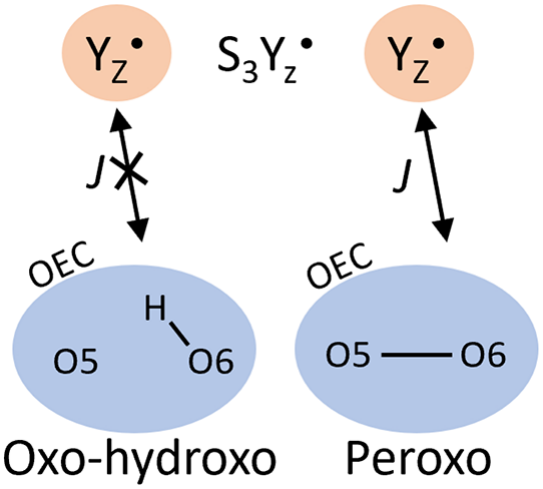

Using BS-DFT (broken-symmetry density functional theory), the electronic and magnetic properties of the S_3_Y_Z_^•^ state of photosystem II were investigated and compared to those of the S_3_ state. While the O5 oxo–O6 hydroxo species presents little difference between the two states, a previously identified [O5O6]^3–^ exhibits reduced stabilization of the O5–O6 shared spin. This species is shown to have some coupling with the Y_Z_^•^ center through Mn_1_ and O6. Similarly, a peroxo species is found to exhibit significant exchange couplings between the Y_Z_^•^ center and the Mn cluster through Mn_1_. Mechanistic changes in O–O bond formation in S_3_Y_Z_^•^ are highlighted by analysis of IBOs (intrinsic bonding orbitals) showing deviation for Mn_1_ and O6 centered IBOs. This change in coupling interactions throughout the complex as a result of S_3_Y_Z_^•^ formation presents implications for the determination of the mechanism spanning the end of the S_3_ and the start of the S_4_ states, affecting both electron movement and oxygen bond formation.

Water oxidation, and subsequent dioxygen formation, is catalyzed by the manganese–calcium oxygen-evolving complex (OEC) held within the photosystem II protein framework.^1−4^ Taking place over a series of steps such that
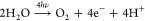
1the mechanism can effectively be broken down into a series of photoactivated oxidation and deprotonation steps with modern efforts, both experimental and theoretical, directed toward the determination of both the order and relative timing of each event.^5−13^ An in-depth understanding of this catalytic cycle is intrinsic in driving the development of future technologies aimed at harnessing both the water-splitting potential of the complex and its capacity to store energy to account for an increasing global energy demand.^14−20^

During water oxidation, the OEC moves through five states (S_0_ to S_4_) determined by the number of oxidizing equivalents stored, signified by the subscript numeral. Of these states, S_0_–S_3_ can be isolated for study, while S_4_ has yet to be isolated and is strongly assumed to be transient. Due to the transient nature of S_4_, increased importance is then placed on the description of S_3_ in order to both understand S_3_ itself and determine viable structures at the beginning of S_4_.

Early crystal structures of the S_3_ state^21,22^ show a short (≈1.5 Å) O–O distance between O5 and O6 (Figure S1) suggesting early onset O–O bond formation in the form of a peroxide or superoxide structure.^22^ However, this idea is excluded when interpreted alongside earlier spectroscopic^23,24^ and later SFX structures,^25,26^ suggesting an O5–O6 distance of ≈2.0 Å and therefore noninteracting or weakly interacting oxygens at the O5 and O6 positions. Recent theoretical work has put forward a comprehensive overview of the potential energy surface as a function of O5–O6 distance throughout the S_3_ state.^5−8^ An oxo–hydroxo O5–O6 formation was presented, in line with previous work in the field,^24,27−30^ transitioning to an oxo–oxyl intermediate close to the geometry of modern crystal structures.^9^ Finally, this proceeds to a peroxo structure as O5–O6 distance is reduced.

In addition to the initial bonding interaction between O5 and O6, the transition from S_3_ to S_4_ also comprises a final oxidation event,^31−35^ during which the local Tyr161 (Y_Z_) is oxidized to a Y_Z_^•^ state. This oxidation is proposed to have occurred within 50 μs after the flash. This is thought to be followed by a deprotonation event with the proton leaving through the Cl1 channel (200–500 μs). This is followed by OEC oxidation (500–1200 μs), Y_Z_^•^ reduction (500–730 μs), and molecular oxygen formation (≈1200 μs).^34,35^ The oxidization event can, in turn, be considered in three discrete phases:^36^ First, there is the oxidation of Y_Z_. This is followed by an extended lag phase during which additional oxidation is not reported. Finally, the resulting Y_Z_^•^ is reduced. This is followed by the release of molecular oxygen and subsequently another water molecule being inserted and deprotonated along with the reformation of the S_0_ state.

Since the initiation of this final oxidation step is thought to mark the formal transition between the S_3_ and S_4_ states, and the subsequent release of the dioxygen molecule, deducing the effect of Y_Z_ reduction on the overall S_3_ PES is a vital step in understanding the transition from a oxo–hydroxo O5–O6 formation to the pre-O_2_ species. In continuation of previously conducted work,^5^ and accounting for the findings put forward by Pushkar et al.^37^ suggesting that the formation of the oxyl may occur during the lag phase, after the oxidation of Y_Z_, but prior to its eventual reduction, we present an investigative comparison of the O5–O6 bond formation potential energy surface both before and after the oxidation of Y_Z_ in an attempt to better understand how the presence of this local Y_Z_^•^ radical species influences the activity of the OEC and the potential role that Y_Z_^•^ formation has on influencing the O–O species formed at each stage of the S_3_ state.

In the S_3_Y_Z_^•^ state the Y_Z_-161 residue is a positive radical, resulting in the presence of an additional unpaired spin center, increasing the total number of possible broken-symmetry (BS) states. It was found that, in order to produce the correct BS solutions in this state, with Y_Z_^•^ allocated a β spin, intuitively flipping the spin on either the C1 or oxygen atom of the Y_Z_^•^ residue proved to be insufficient, instead requiring both atoms be flipped or indeed all carbon atoms in the Y_Z_^•^ ring as well as the oxygen (see Figure 1). Flipping just C1 (see Figure 1) which carries a majority of the unpaired spin led to problems in the convergence of the wave function for many BS states, as such data for comparison with Figure 1A could not be obtained. Similarly flipping just the Y_Z_^•^ oxygen yielded the incorrect Mulliken spin distribution, with the Y_Z_^•^ remaining in the high-spin state. Instead the spin shared between O5 and O6 was found to be flipped. As a result of this, any BS state calculated with the incorrect spin flips will not produce the desired spin distribution and as such will compromise both the resulting BS energy and the calculated exchange couplings between spin centers.

**Figure 1:**
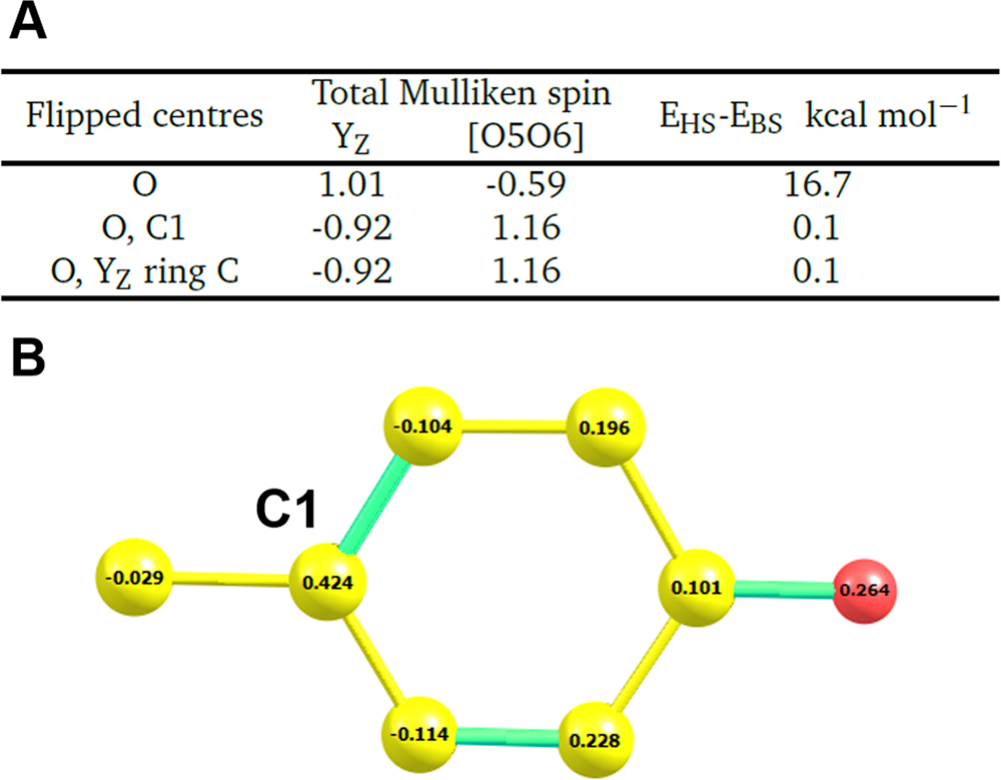
Taken from a high-spin O5–O6 = 2.1 Å model. (A) Total calculated Mulliken spin population for the Y_Z_ residue for different BS “flipspin” inputs, as well as the relative energies compared to the high-spin system. (B) Mulliken spin populations of the Y_Z_^•^ residue. Yellow: carbon. Red: oxygen. Hydrogens are omitted for clarity.

In the S_3_ state the Y_Z_ residue was found to be protonated and hydrogen bonded to its hydrogen bonding partner His190, whereas upon generation of the S_3_Y_Z_^•^ state the proton from the Y_Z_^•^ residue moved onto the His190 residue, leaving the Y_Z_^•^ oxygen deprotonated. It was found that this movement occurred spontaneously and optimization with the proton on either Y_Z_^•^ or His190 resulted in the same geometry, with His190 bearing the proton, leaving the Y_Z_^•^ oxygen as a deprotonated phenoxyl. This suggests barrier-less proton transfer or a negligible barrier for proton movement upon oxidation, rather than a separate discrete proton transfer event. Models with O5 and O6 in a peroxo or oxo–oxo arrangement (*r*[O5O6] = 1.4 or 2.4 Å respectively) were created with the proton fixed on Y_Z_ or His190 to investigate the energy difference for various BS states as well as the HS (high-spin) state. It was found that the peroxo-like arrangement structures with the proton fixed on Y_Z_ were on average 22 kcal mol^–1^ higher in energy than the His protonated counterpart. In the oxo–oxo arrangement, this was increased to 26 kcal mol^–1^. The Y_Z_^•^ residue remains hydrogen bonded through the phenoxyl oxygen to the OEC through the calcium bound W4; this was not observed to change the position or bonding nature significantly when the protonation state of the Y_Z_ residue changed. Similarly the Y_Z_ residue can also hydrogen bond through a nearby water molecule indirectly to the OEC by several pathways depending on proton orientation, but again the position and orientation of this water molecule was also unaffected by the protonation state.

Previous work in the S_3_ state investigated several potential energy surfaces (PESs) resulting in the identification of three key species:^5^ an O5 oxo–O6 hydroxo; a [O5O6]^3–^ with a single unpaired β spin shared between O5 and O6; and a peroxo species with a formal bond between O5 and O6. Once the Y_Z_ residue is oxidized and the S_3_Y_Z_^•^ state formed there is an additional spin center present. For the O5 oxo–O6 hydroxo there are now 5 spin centers (Mn_1_–Mn_4_ and Y_Z_) yielding 15 unique BS states (and 1 HS state), whereas in the S_3_ state, there are only 7 unique BS states. The relative energies of these are compared in Figure 2. The most stable BS states are either with Mn_4_ or Mn_3_ antiparallel to all other spin centers regardless of the Y_Z_^•^ spin alignment or whether the model is in the S_3_ state or S_3_Y_Z_^•^ state. Furthermore, the alignment of the Y_Z_^•^ radical has no significant effect on the BS energies in the S_3_Y_Z_^•^ state. This, in turn, suggests a lack of, or weak coupling between, the Y_Z_^•^ radical and the Mn cluster; this aligns with previous work by Retegan et al.^38^ in which the S_2_Y_Z_^•^ showed no coupling between the Y_Z_^•^ residue and the OEC.

**Figure 2:**
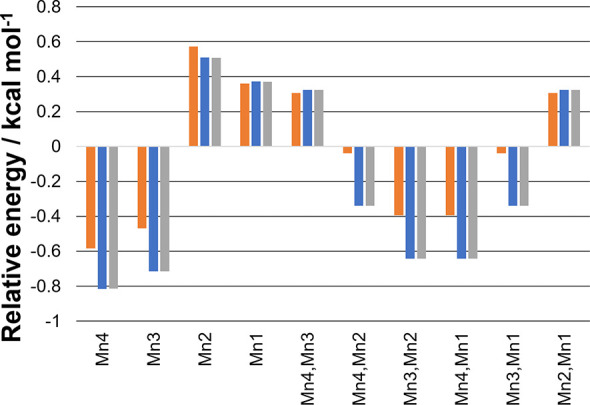
Relative energy of the BS states to the HS state for the oxo–hydroxo models: more negative values correspond to more stable BS states. The “flipped” centers are indicated. Orange: S_3_ state. Blue: S_3_Y_Z_^•^ state with Y_Z_^•^ as α. Gray: S_3_Y_Z_^•^ state with Y_Z_^•^ as β.

The lack of coupling of the oxo–hydroxo OEC with the nearby Y_Z_^•^ radical observed in Figure 2 is reflected in the calculated *J* values shown in Table 1. Comparing the S_3_ and S_3_Y_Z_^•^*J* values, it can be seen that the magnitude is similar for all Mn–Mn couplings. There is also an apparent lack of significant coupling between the Mn centers and the Y_Z_^•^ residue, and the coupling here is several orders of magnitude smaller. This agrees with previous findings by Retegan et al.^38^ who reported a similar difference in magnitude for the S_2_Y_Z_^•^ state.^38^ The dominant antiferromagnetic coupling between Mn_3_ and Mn_4_ agrees well with the BS4 and BS3 states being the most stable.

**Table 1:** BS-DFT Calculated Mn–Mn, Mn–Y_Z_, Mn–O, and O–Y_Z_^•^ Exchange Couplings, *J* Values Obtained for the O5 Oxo–O6 Hydroxo, the Broad Minima Corresponding to [O5O6]^3–^ and the O5–O6 Peroxo State, in Both the S_3_ and S_3_Y_Z_^•^ State^a^

	oxo–hydroxo		[O5O6]^3–^		peroxo	
	S_3_Y_Z_^•^	S_3_	S_3_Y_Z_^•^	S_3_	S_3_Y_Z_^•^	S_3_
*J*_43_	–35.6	–26.1	–7.7	–17.8	21.4	17.8
*J*_42_	0.6	0.4	–0.6	0.6	1.8	1.2
*J*_41_	3.7	3.2	–39.6	–87.7	–3.7	–5.8
*J*_4–O5/O6_			–536.9	–1051.7		
*J*_4*Y*_Z__	0.0		–4.1		–10.1	
*J*_32_	8.3	9.4	17.5	11.3	11.1	10.1
*J*_31_	–0.1	–1.4	–12.8	–25.7	–4.2	–6.7
*J*_3–O5/O6_			–342.1	–432.8		
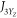	0.0		–3.9		4.9	
*J*_21_	10.6	12.0	12.6	14.1	–30.8	–33.5
*J*_2–O5/O6_			0.5	3–0.2		
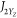	0.0		0.0		–11.1	
*J*_1–O5/O6_			–849.9	–1301.1		
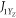	0.0		1.3		–27.6	
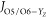			–16.4			

a All values are in cm^–1^.

During O5–O6 bond formation it is necessary to deprotonate O6. At a large O5–O6 separation (>2.2 Å) this yields an oxo–oxo species while optimization at a short O5–O6 distance (<1.6 Å) yields a peroxo structure with O5 and O6 bonded. Optimization at around 2 Å yields a [O5O6]^3–^ species both in the S_3_ and S_3_Y_Z_^•^ state. In both states an unpaired β electron is shared between O5 and O6 while other spin centers are in an α alignment; a spin density plot for the S_3_Y_Z_^•^ state is shown in Figure S3 and illustrates both this and the delocalization of the radical throughout the Y_Z_^•^ residue.

The BS-DFT energies for the [O5O6]^3–^ species are shown in Figure 3 where for any given BS state the stabilization is reduced in the S_3_Y_Z_^•^ state in contrast to the increased stabilization observed in the oxo–hydroxo geometry (Figure 2). Interestingly, the spin alignment of the Y_Z_^•^ as for the O5 oxo–O6 hydroxo has no obvious effect on the energies of most BS states. However, very small differences can be observed for some states, such as the BS1 state. The most stable BS state for both S states is an antiparallel alignment of the [O5O6] shared spin to all other spin centers as expected given the relative magnitudes of the Mn–O_5/6_*J* couplings compared to the Mn–Mn and Y_Z_–O_5/6_*J* couplings (Table 1). Given the relative magnitudes of the [O5O6] couplings, this strongly marked effect may overshadow the more subtle remaining couplings throughout the system.

**Figure 3:**
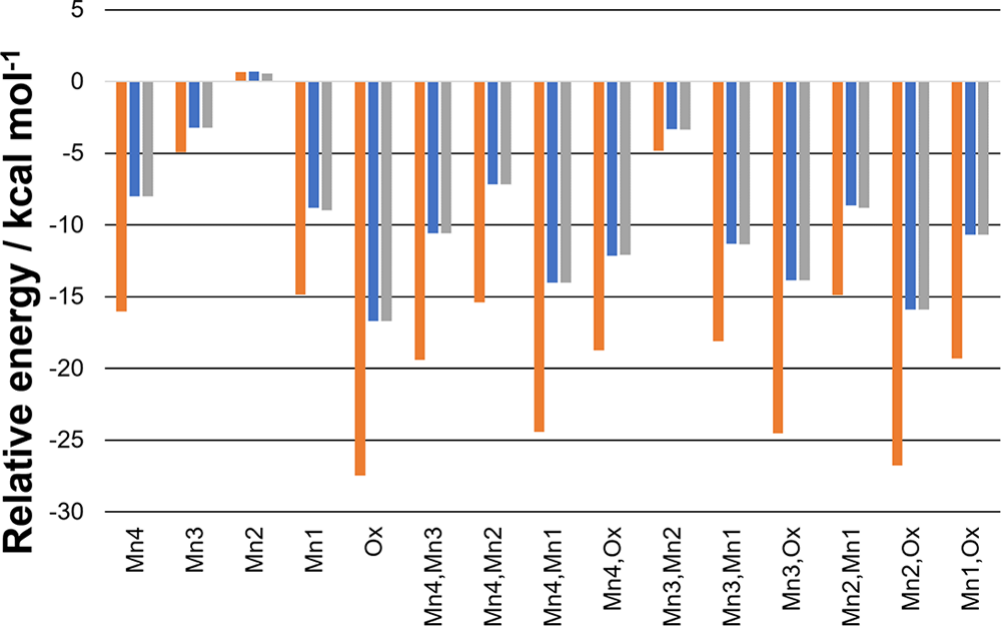
Relative energy of the BS states to the HS state for the [O5O6]^3–^ species; more negative values correspond to more stable BS states. The “flipped” centers are indicated. Orange: S_3_ state. Blue: S_3_Y_Z_^•^ state with Y_Z_^•^ as α. Gray: S_3_Y_Z_^•^ state with Y_Z_^•^ as β.

The [O5O6]^3–^ HS form with the shared spin between the oxygens antiparallel is energetically the most favored (see Table S1); it is also clear from Table 1 that it is the Mn–O5/O6 couplings which dominate the spin interactions. As such, the relative reduced stability of the [O5O6]^3–^ species in the S_3_Y_Z_^•^ state could be rationalized by the decreased stabilization of the shared spin by the Mn centers. Furthermore, in the S_3_Y_Z_^•^ state, coupling between the Y_Z_^•^ spin and the OEC is observed, particularly between the shared oxygen spin and Y_Z_^•^ (see Table 1). From Table 1 it can be seen that in both states the spin shared between O5 and O6 is stabilized by strong antiferromagnetic coupling with the Mn_1_, Mn_3_, and Mn_4_ centers. These couplings are significantly larger than all other couplings and so rationalize why this center dominates the BS energies. In contrast, the coupling between Mn_2_ and O5 and O6 is negligible. This is likely due to the fact that O5 and O6 are directly bonded to Mn_1_, Mn_3_, and Mn_4_ but not to Mn_2_, therefore preventing the strong coupling observed with the other metal centers. This observation is further backed up by the low coupling between Mn_2_ and Mn_4_ when compared to stronger coupling with Mn_1_ and Mn_3_. This stabilizing coupling with the Mn ions is somewhat diminished in the S_3_Y_Z_^•^ state compared to the S_3_ state with Mn–[O5O6] couplings decreasing on average by a factor of 1.6, rationalizing why the BS-DFT energies in Figure 3 are reduced in the S_3_Y_Z_^•^ state. Interestingly, some antiferromagnetic coupling is also observed between [O5O6]^3–^ and the Y_Z_^•^ radical comparable in magnitude to the Mn–Mn couplings, showing the presence of couplings between the OEC and the Y_Z_ residue. Similarly, although weaker, the Mn ions also show coupling with the Y_Z_^•^ spin. Comparing the S_3_ and S_3_Y_Z_^•^ state, the most significant changes in magnitude are observed for couplings involving Mn_1_ and O5/O6.

At short O5–O6 separation the OEC optimized to a peroxo type structure, with a formal O–O bond between O5 and O6. For this species, the BS1 state was found to be the lowest in energy. This peroxo geometry shows a much more significant coupling between the OEC and the Y_Z_^•^ radical. The calculated *J* values for the peroxo species both in the S_3_ and S_3_Y_Z_^•^ state are shown in Table 1 and the BS energies in Figure 4. While the Mn–Mn couplings are very similar for the S_3_ and S_3_Y_Z_^•^ state, significant coupling between the Mn centers and the Y_Z_^•^ radical are observed, the effect of which is reflected in the BS energies, despite the comparatively large separation of the OEC and the Y_Z_^•^ radical. The Y_Z_^•^ radical shows antiferromagnetic coupling with Mn_4_, Mn_2_, and Mn_1_ and shows weak ferromagnetic coupling with Mn_3_, with the strongest coupling observed with the Mn_1_ center. Mn_3_ is shielded from Y_Z_^•^ by Mn_2_ and Ca^2+^ rationalizing the weaker coupling. The BS energies indicate the most stable BS state to be BS1 with all other centers antiparallel to it. This agrees well with the strong antiferromagnetic Mn_1_–Y_Z_^•^ coupling and indeed Mn_1_–Mn_2_.

**Figure 4:**
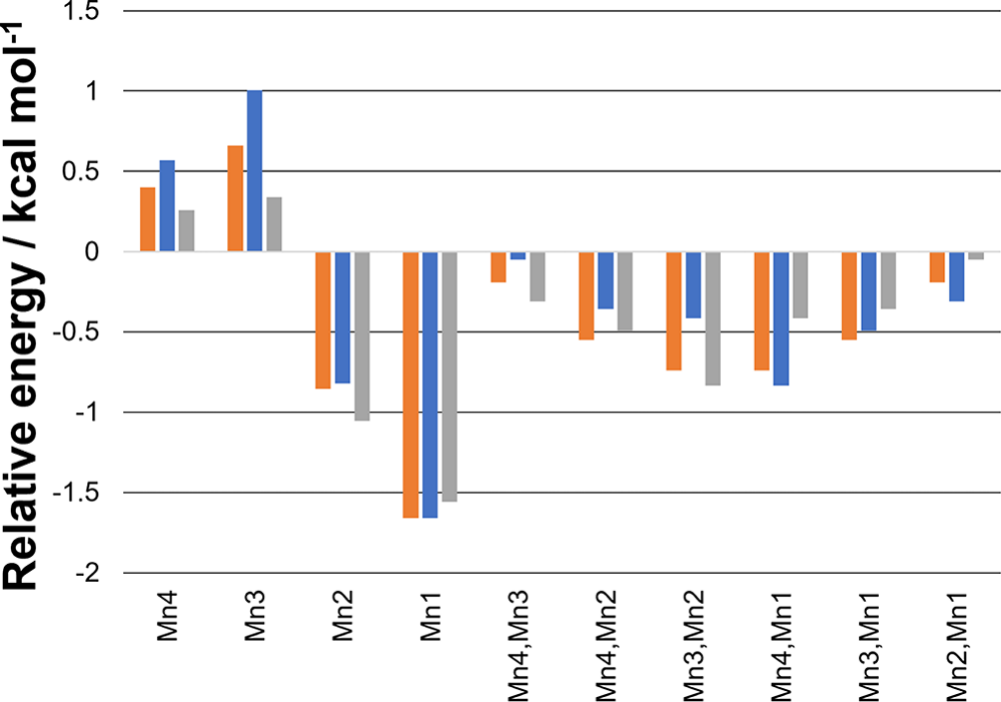
Relative energy of the BS states to the HS state for the peroxo species: more negative values correspond to more stable BS states. The “flipped” centers are indicated. Orange: S_3_ state. Blue: S_3_Y_Z_^•^ state with Y_Z_^•^ as α. Gray: S_3_Y_Z_^•^ state with Y_Z_^•^ as β.

Investigating the electronic changes by analysis of the intrinsic bonding orbitals (IBOs) in the S_3_Y_Z_^•^ state for the partial O–O bond formation of the [O5O6]^3–^ shows no major differences between the S_3_ and S_3_Y_Z_^•^ state for the high-spin *M_s_* = 6.5 oxo–oxo/peroxo surface, see Supporting Information Figures S4 and S5. Here the only apparent difference is the π bonding lone pair on O6 (magenta) for which the spin orbital’s orientation differs; in the S_3_ state, the spin orbital lies along the Mn_1_–O6–Mn_3_ plane, whereas in the S_3_Y_Z_^•^ state it is perpendicular to it.

For the BS1 oxo–oxo/peroxo surface (*M_s_* = 3.5) (Figure 5), 5 IBOs were observed to change significantly across the PES, as opposed to only four in the S_3_ state (see Figure S6). An α electron from the lone pair on O6 (green) that shows some π bonding character to Mn_1_ forms the α component of the O5–O6 bond around 2 Å. Also, an α electron from a Mn_4_–O5 σ bond (blue) moves to Mn_4_ around 2 Å, while the corresponding β electron from the Mn_4_–O5 σ bond (red) moves to form the β component of the O5–O6 bond around 1.8 Å. These three IBOs and their changes are the same for both the S_3_ and the S_3_Y_Z_^•^ state. However, the remaining two IBOs differ: In the S_3_ state a β electron from a Mn_1_–O6 σ bond moved onto Mn_1_ around 1.8 Å with an associated orbital change of 1 e^–1^ (see Figure S6), whereas here, in the S_3_Y_Z_^•^ state, the β electron from a Mn_1_–O6 σ bond (yellow) moves to become a lone pair β on O6 around 1.8 Å with an associated orbital change of 0.5 e^–1^ (see Figure S7). Finally, a lone pair β electron (magenta), which shows no interaction with Mn_1_ initially, unlike the corresponding α electron (green), moves to Mn_1_ around 1.8 Å with an associated orbital change of 1.5 e^–1^. These two changes still amount to an overall change of 1 e^–1^ going toward Mn_1_ but present different sources for the observed change.

**Figure 5:**
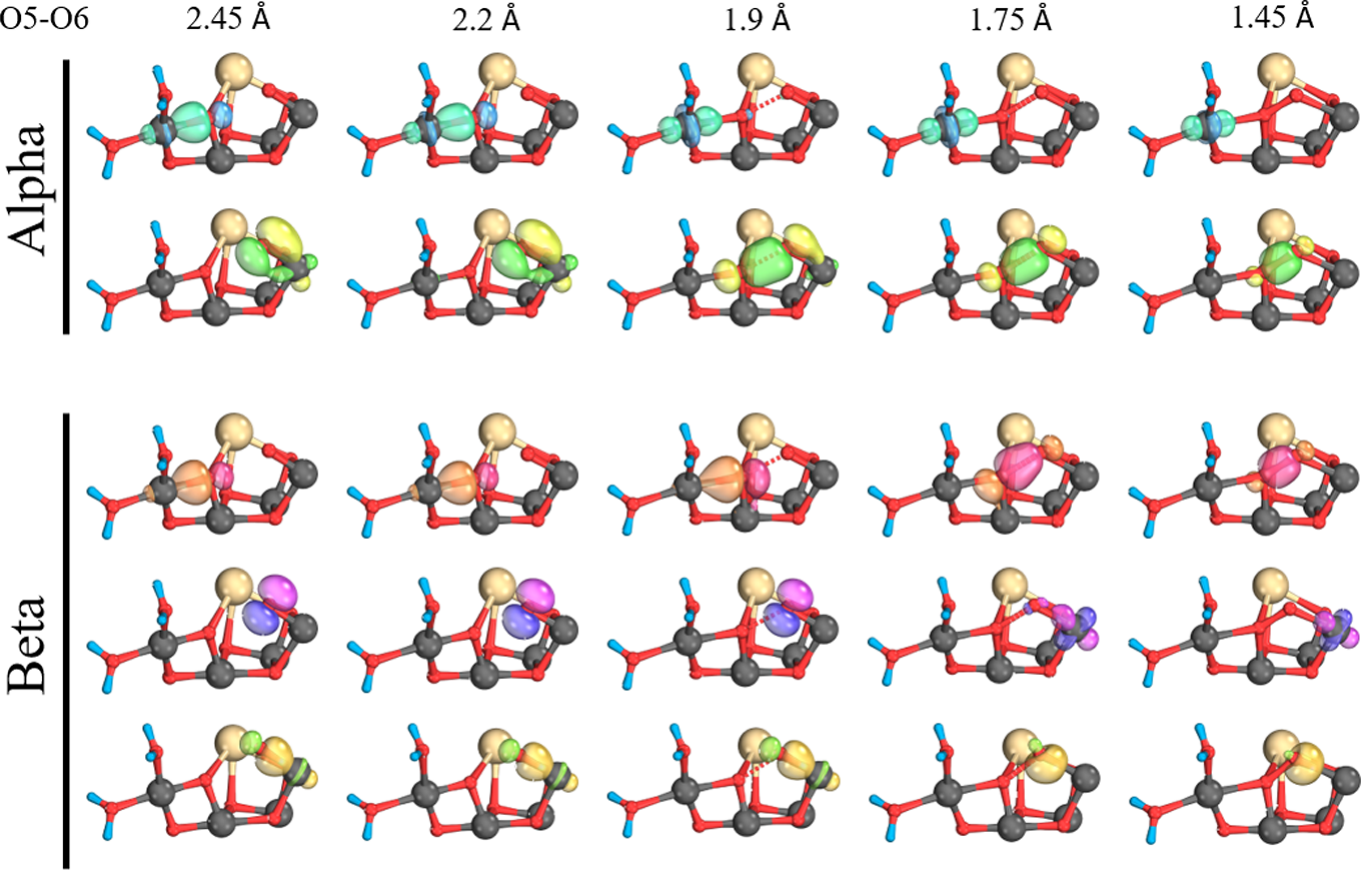
Intrinsic bond orbital (IBO) analysis of the *M_s_* = 3.5 state oxo–oxo form. IBOs are given at the indicated O5–O6 separations showing α and β spin evolution.

While at first glance a long-range spin–spin interaction such as the coupling between the OEC and the Y_Z_^•^ residue seems unlikely, long-range spin–spin couplings are well established in the literature for both organic and metallic spin centers.^39−42^ Within the OEC, the spin–spin couplings have been shown to be superexchange type interactions,^43^ relying on the oxygen bridges between Mn ions. While superexchange through space is possible, in examples studied by Stanford et al.^41^ it was shown that favorable orbital overlap is required; it has also been shown that long-range superexchange can exist in certain systems containing two spin centers linked by covalent bonds.^42^ Additionally, solvent molecules may mediate coupling interactions between spin centers and can aid in electron transfer mechanisms.^44−46^ Liu et al.^40^ demonstrated the presence of long-range coupling between two spin centers, linked by a flexible linker which did not allow for delocalization, and showed that increasing linker length did not always have an effect on the magnitude of the spin–spin coupling. For electron transfer between interacting spin systems it has been found that the relative orientation of the two spin centers can be important for electron transfer and that the strength of the coupling is proportional to the rate of electron transfer as described by Fermi’s golden rule.^47−50^ Observation of coupling between the OEC and the Y_Z_^•^ residue may point toward the potential for efficient electron transfer between them. Furthermore, the relative strengths of the couplings between the various spin centers in the OEC with the Y_Z_^•^ suggest the origin of the electron transfer pathway to be through either [O5O6]^3–^ or Mn_1_, or indeed through both, to the Y_Z_^•^ residue. Identifying the nature of the electron transfer process between the OEC and Y_Z_^•^ is crucial, as this is a key step in the transition between the S_3_ and S_4_ state.

The S_3_Y_Z_^•^ state is an intermediate S-state, and so far it has not been possible to isolate it, limiting the amount of experimental data for it. Nonetheless some data is available; for example, EPR detects a split signal when the S_3_ or indeed S_0–3_ is illuminated by light of a specific wavelength. This has been attributed to the presence of a Y_Z_^•^ radical.^51^ The ground state spin between the S_3_ and S_3_Y_Z_^•^ state intermediates differs; in the S_3_ the calculated ground state spin for the oxo–hydroxo, [O5O6]^3–^, and peroxo species are 3, 6, and 3, respectively, whereas in the S_3_Y_Z_^•^ state they were 3.5, 5.5, and 3.5, respectively. As such, EPR should be able to differentiate between them. To the best of the authors’ knowledge, however, so far the ground state spin of the species in the S_3_Y_Z_^•^ state has not been determined. XFEL studies have shed some light on the sequence of events for the S_3_ to S_0_ state;^34,35^ these suggest that the Y_Z_^•^ is present until O6 disappears (suggesting O_2_ formation), in line with the mechanism proposed here in which the peroxo species is oxidized to reduce the Y_Z_^•^ radical forming superoxo, which would rapidly form molecular oxygen.

Both the [O5O6]^3–^ species and the peroxo species presented here showed significant exchange coupling interactions between the Y_Z_^•^ radical and the OEC through the shared spin of Mn_1_ and [O5O6] shared spin. On the other hand the oxo–hydroxo species showed no exchange interaction agreeing well with previous S_2_ state work.^38^ The species differ by both the O5–O6 separation as well as the O6 protonation state and orbital orientations, as seen for the [O5O6]^3–^ species where the O6 lone pair was found to point toward the Y_Z_^•^ radical in the S_3_Y_Z_^•^ state but not in the S_3_ state. For the [O5O6]^3–^ species, antiferromagnetic coupling between the Y_Z_^•^ radical and O5/O6 was observed along with weaker coupling between Y_Z_^•^ and the Mn ions. Weaker couplings between Mn_1_ and the remaining Mn ions, and reduced [O5O6]–Mn couplings when compared to the S_3_ state, suggest that the [O5O6]^3–^ species is relatively destabilized in the S_3_Y_Z_^•^ state compared to the S_3_ state agreeing with the relative energetics of the BS states.

Similarly, for the peroxo species, large exchange couplings were observed between the Y_Z_^•^ radical and the Mn ions, with particularly strong coupling observed with Mn_1_. An additional IBO was observed to change significantly in the S_3_Y_Z_^•^ state, and two IBOs, both involving Mn_1_ and O6, showed major differences in their behavior when compared to the S_3_ state, further pointing toward long-range exchange coupling between the Y_Z_^•^ radical and the OEC. This would suggest potential facilitation of electron transfer from OEC to Y_Z_^•^ through the coupled [O5O6], Mn_1_, and Y_Z_^•^ spin centers.

Throughout the Kok cycle, Y_Z_ subsequentially removes electrons from the OEC, preparing the system for facile molecular oxygen evolution. As the O5–O6 bond is formed, increased exchange coupling between the Y_Z_^•^ radical and the OEC has been shown to emerge. The presence of this exchange coupling between the OEC and Y_Z_^•^ may suggest a mechanism through which removal of the final electron from the OEC is promoted. The removal of the final electron would aid to drive the catalytic cycle to completion by promoting the onward reaction of peroxo. Due to the more stable nature of peroxo, it is reasonable to assume that additional driving force would be highly beneficial to this particular oxidation step.

## Methods

The methods used are similar to those described previously.^5,6,43^ All calculations were performed in ORCA 4.^52^ Models were initially optimized using the B3LYP functional^53,54^ in their HS oxidation states. The zeroth-order regular approximation (ZORA) Hamiltonian was applied to account for scalar relativistic effects^55−57^ with the def2-SVP basis sets used for C and H atoms and the def2-TZVP basis set without f functions for all other atoms.^58^ For the systems presented here the B3LYP functional was chosen as it has been shown to work well for systems of this size and for energetics and orbital analysis for the OEC^59^ as well as other transition metal systems.^60^ The chain of spheres (RIJCOSX) approximation was applied together with the decontracted general Weigend auxilary basis sets.^61−65^ The conductor-like polarizable continuum model (CPCM) with a dielectric constant of ϵ = 8.0 was used throughout to model the protein environment,^66,67^ along with the dispersion corrections proposed by Grimme with Becke–Johnson damping (D3BJ).^68,69^ Tight SCF convergence criteria and increased integration grids (Grid6 and IntAcc 6 in the ORCA convention) were used throughout, and all terminal carbon atoms were constrained during optimizations.

Initial BS-DFT wave functions were calculated using ZORA versions of the def2-TZVP with removed f functions for all atoms, and used for potential energy surface calculations.^58^ The initial BS guesses were obtained by use of the “flipspin” feature of ORCA.^70^ Also, convergence of the correct BS and HS states was confirmed by examination of the calculated Mulliken spin populations for all calculations. Throughout the text, these BS states may be referred to as BS1 or BS4, for example, indicating a BS state with Mn_1_ or Mn_4_ flipped, respectively.

The potential energy surface was obtained using the geometry optimization method as described above. The BS “.gbw” file for the *M_s_* state in question was initially read in, and the O5–O6 bond length was varied between 2.45 to 1.45 Å in 0.05 Å steps, where each point underwent full geometry optimization to produce the final potential energy surface. To investigate the relative energies of His190 and Y_Z_ protonated structures, a high-spin O5–O6 peroxo or high-spin O5 oxo–O6 oxo was optimized. The proton was then moved onto either His190 or Y_Z_, and single point calculations were performed for various BS states. Intrinsic bond orbitals (IBOs) were produced using IboView with iboexp = 2 from the optimized PES wave functions.

All models were generated from the S_3_ XFEL crystal structure (PDB: 6DHO)^9^ and optimized in the S_3_Y_Z_^•^ state. Seven directly coordinated amino acids are included in the models. Six are from the D1 protein chain (Asp170, Glu189, His332, Glu333, Asp342, Ala344, Tyr161 (Y_Z_), and His190) and one from the CP43 protein chain (Glu354). The second sphere His337 residue was included along with the partial backbone of Ser169 (Figure S2). Terminal carbon atoms were constrained in all calculations. The directly coordinated water molecules W1–W4 as well as 11 crystallographic water molecules were also included. All oxygen bridges O1–O5 were in their fully deprotonated form (O^2–^); O6 was ^–^OH for the O5 oxo–O6 hydroxo models, and was O^2–^ otherwise. W1, W3, and W4 were fully protonated, and W2 was ^–^OH for the O5 oxo–O6 hydroxo models, and fully protonated otherwise.

## Supporting Information Available

Supplementary IBOs and structural illustration (PDF)
