# Supplementary material for: Insights into PSII’s S3YZ• State: An Electronic and Magnetic Analysis
Source: J Phys Chem Lett. 2024 Jan 8;15(2):499–506. doi: 10.1021/acs.jpclett.3c03026 (PMC10801681; doi:10.1021/acs.jpclett.3c03026)
Supplement: Supplementary file 1 — jz3c03026_si_001.pdf [file jz3c03026_si_001.pdf]

Insights into PSII's $S_3Y_Z^{\bullet}$ State; an Electronic and Magnetic Analysis

Felix Rummel,† Thomas Malcomson,*,‡ Maxim Barchenko,† and Patrick J. O'Malley†

†*Department of Chemistry, School of Natural Sciences, The University of Manchester, Manchester, M13 9PL, UK.*

‡*School of Biosciences, Cardiff University, Museum Avenue, Cardiff, CF10 3AX, UK.*

E-mail: malcomsonT@cardiff.ac.uk

Table S1: BS-DFT relative energies of the HS optimised geometry presented as $E_{HS}-E_{BS}$ in kcal mol^{-1} for the S_3 state and the $S_3Y_Z^{\bullet}$ state, in both cases using the high spin O5 oxo-O6 hydroxo form as the reference point.

$E_{HS}-E_{BS}$ / kcal mol^{-1}		S_3				$S_3Y_Z^{\bullet}$			
	BS-State	oxo-hydroxo	$[O5O6]^{3-}$		peroxo	oxo-hydroxo	$[O5O6]^{3-}$		peroxo
			α	β			α	β	
Y_Z (α) if applicable	αααα (HS)	0.0	-34.4	-6.8	-29.5	0.0	-41.5	-24.8	-34.9
	βααα	-0.4	-19.4	-15.0	-27.9	-0.4	-32.7	-30.8	-33.2
	αβαα	-0.6	-35.0	-7.5	-28.7	-0.5	-42.2	-25.6	-34.1
	ααβα	0.5	-29.4	-9.8	-30.2	0.7	-38.3	-27.6	-35.9
	αααβ	0.6	-18.3	-15.6	-29.9	0.8	-33.5	-29.3	-35.4
	ββαα	-0.3	-19.4	-14.9	-29.4	-0.3	-32.8	-30.9	-34.6
	βαβα	0.0	-16.2	-18.9	-29.0	0.3	-30.2	-34.3	-34.4
	βααβ	0.4	-9.9	-29.5	-28.8	0.6	-27.5	-38.1	-34.0
Y_Z (β) if applicable	αααα	N/A				0.0	-41.3	-24.8	-34.4
	βααα					-0.4	-32.5	-30.8	-33.3
	αβαα					-0.5	-42.0	-25.6	-33.8
	ααβα					0.7	-38.2	-27.6	-35.2
	αααβ					0.8	-33.5	-29.4	-35.1
	ββαα					-0.3	-32.7	-30.9	-34.8
	βαβα					0.3	-30.1	-34.3	-34.5
	βααβ					0.6	-27.5	-38.1	-34.5

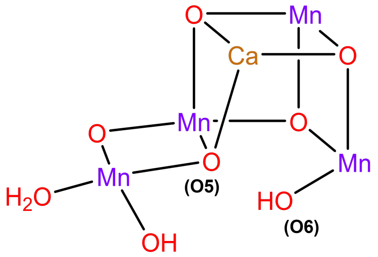

Figure S1: Structure of the OEC at the beginning of the S3 state.

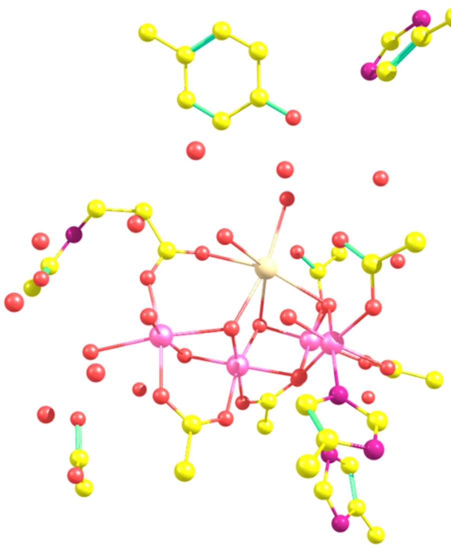

Figure S2: Example of model used for all PES calculations. Hydrogens have been excluded for clarity. Colour coding: Mn (purple), oxygen (red), calcium (cream), carbon (yellow) and nitrogen (purple).

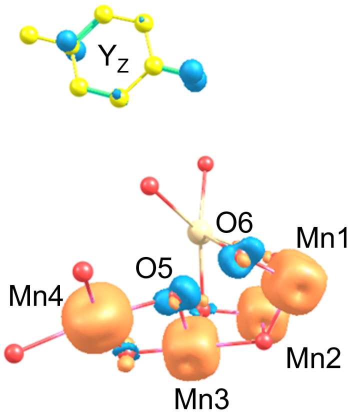

Figure S3: Spin density contour plot for the $[O5O6]^{3-}$ species at 2.05 Å O5-O6 separation, negative spin (blue), positive spin (orange), O5 and O6 share spin, confirming the presence of $[O5O6]^{3-}$.

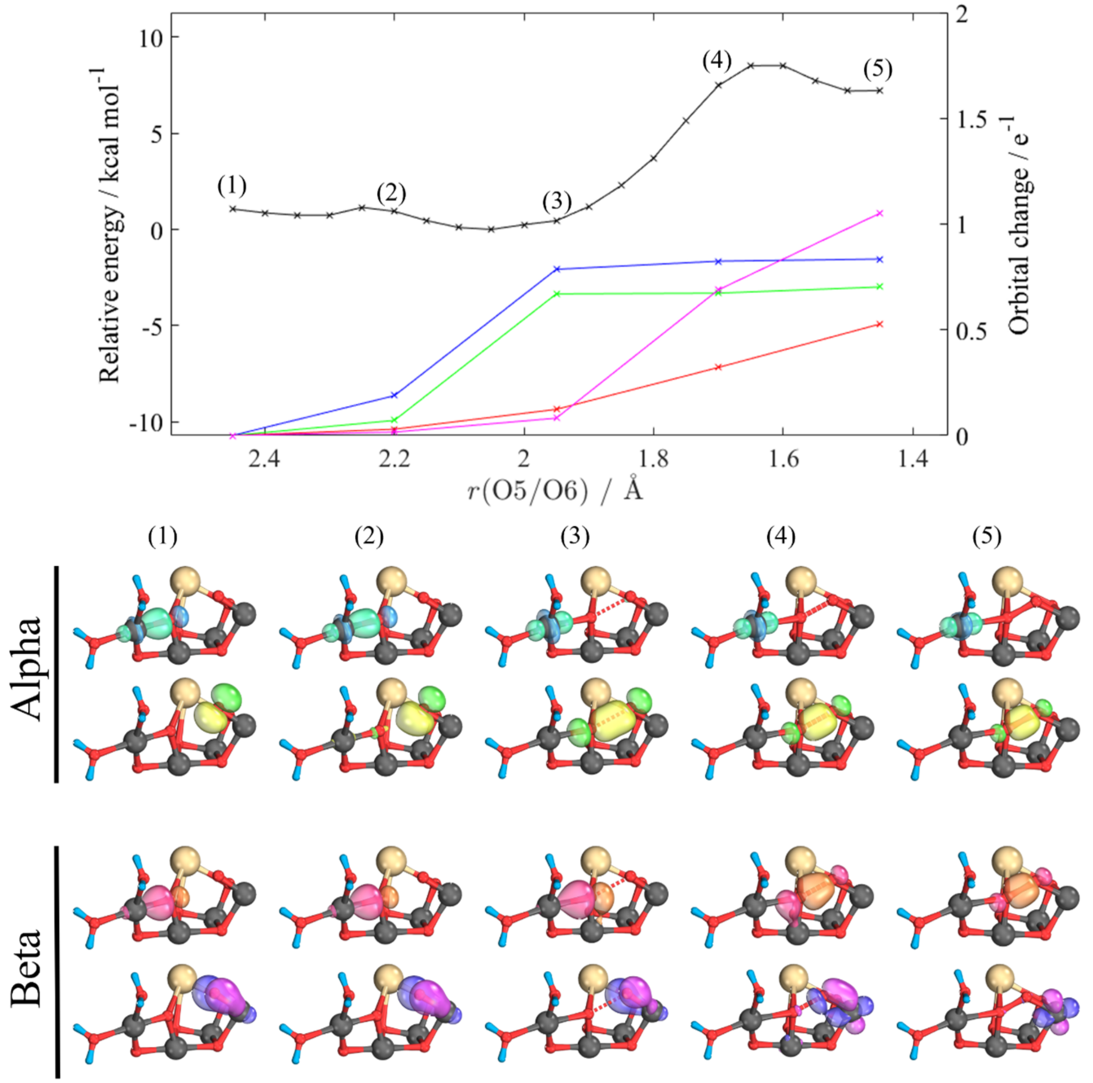

Figure S4: Intrinsic bond orbital (IBO) analysis of the $Ms = 6.5$ state of the oxo-oxo form in the $S_3Y_Z^{\bullet}$ state. Top, potential energy surface (PES) for O5-O6 bond formation (black) with corresponding IBO changes shown beneath, colour coded by orbital. IBOs are given at the labelled points on the PES showing α and β spin evolution.

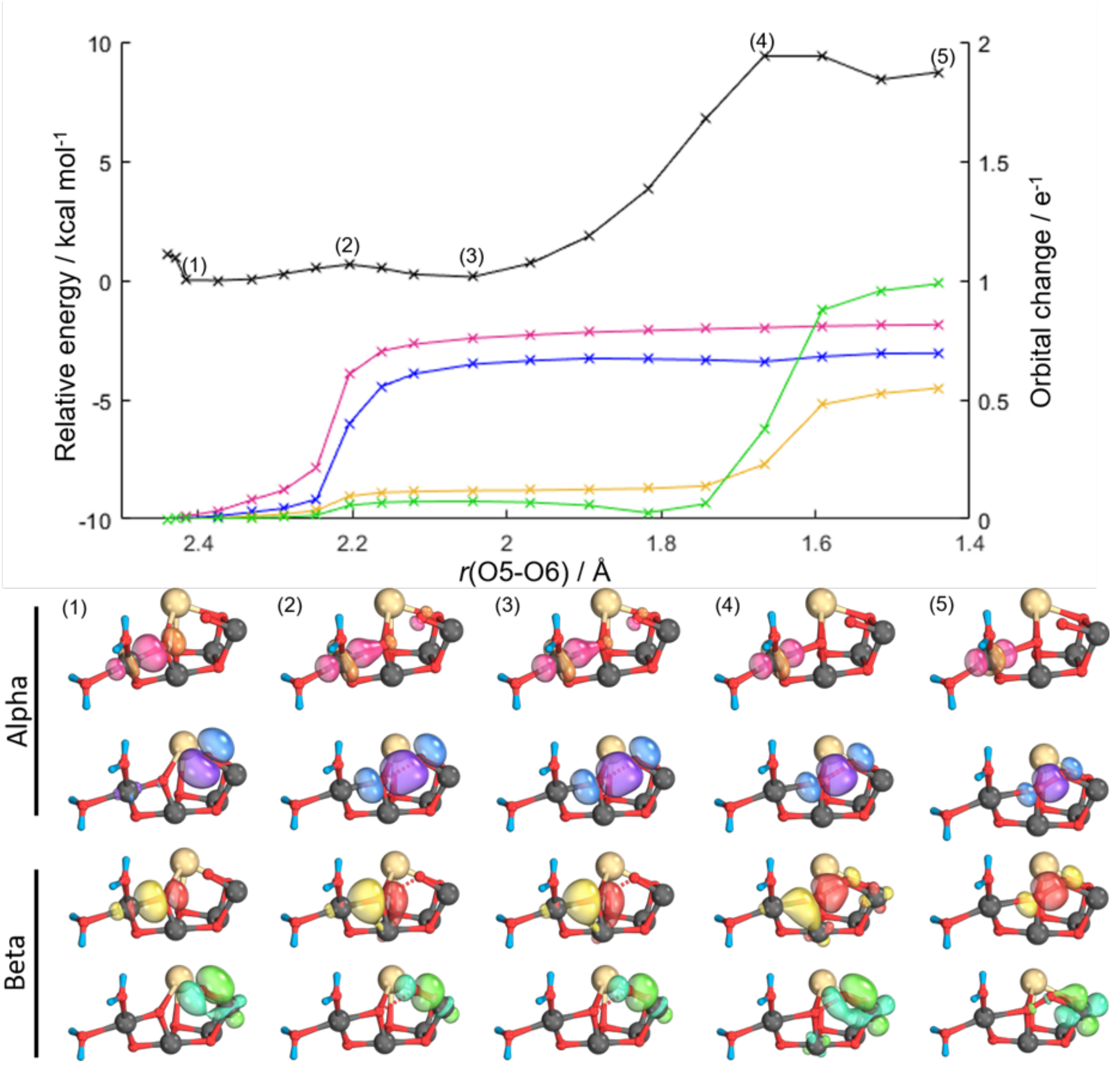

Figure S5: Intrinsic bond orbital (IBO) analysis of the $Ms = 6$ state of the oxo-oxo form in the S_3 state. Top, potential energy surface (PES) for O5-O6 bond formation (black) with corresponding IBO changes shown beneath, colour coded by orbital. IBOs are given at the labelled points on the PES showing α and β spin evolution. Reproduced from Rummel et al.[1]

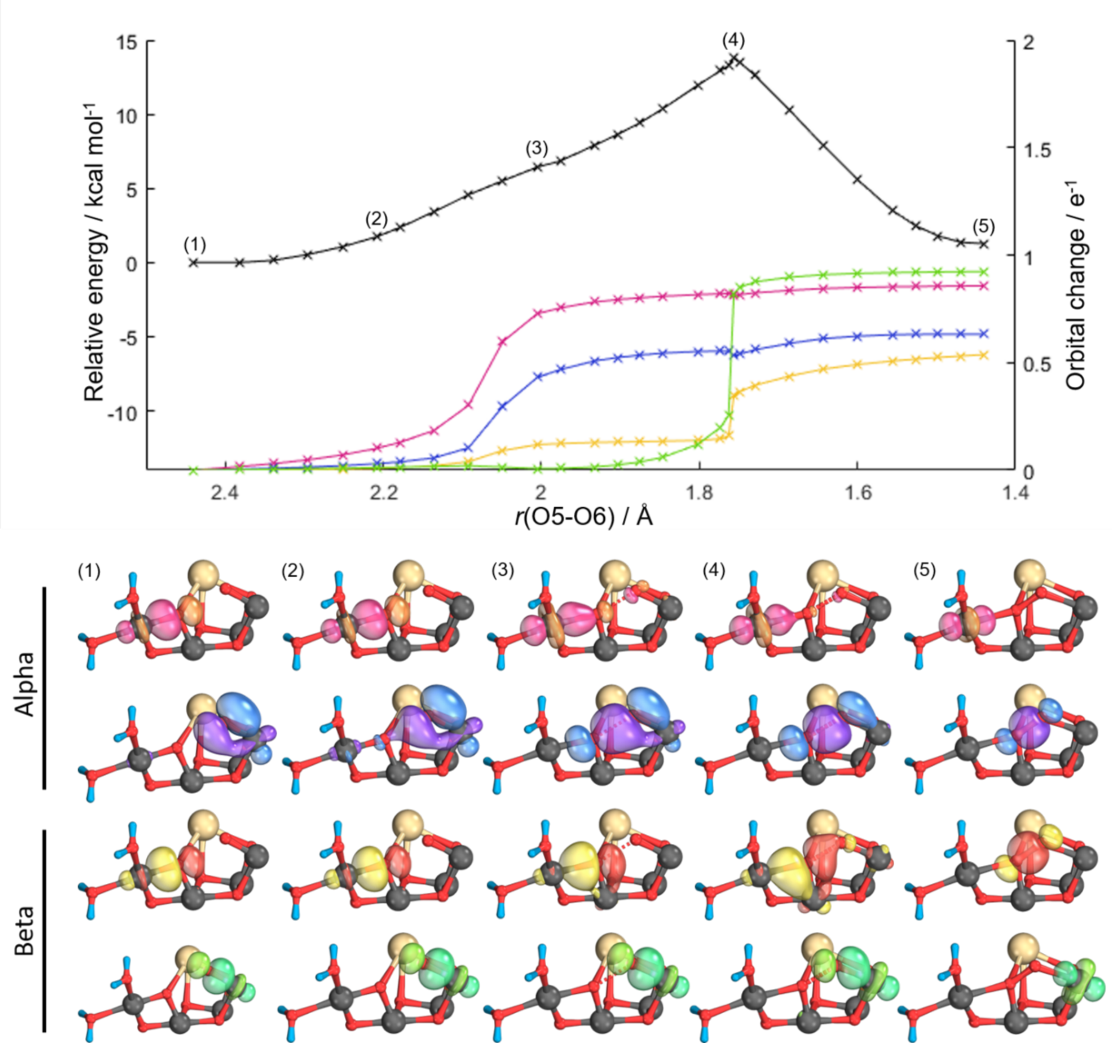

Figure S6: Intrinsic bond orbital (IBO) analysis of the $Ms = 3$ state of the oxo-oxo form in the S_3 state. Top, potential energy surface (PES) for O5-O6 bond formation (black) with corresponding IBO changes shown beneath, colour coded by orbital. IBOs are given at the labelled points on the PES showing α and β spin evolution. Reproduced from Rummel et al.[1]

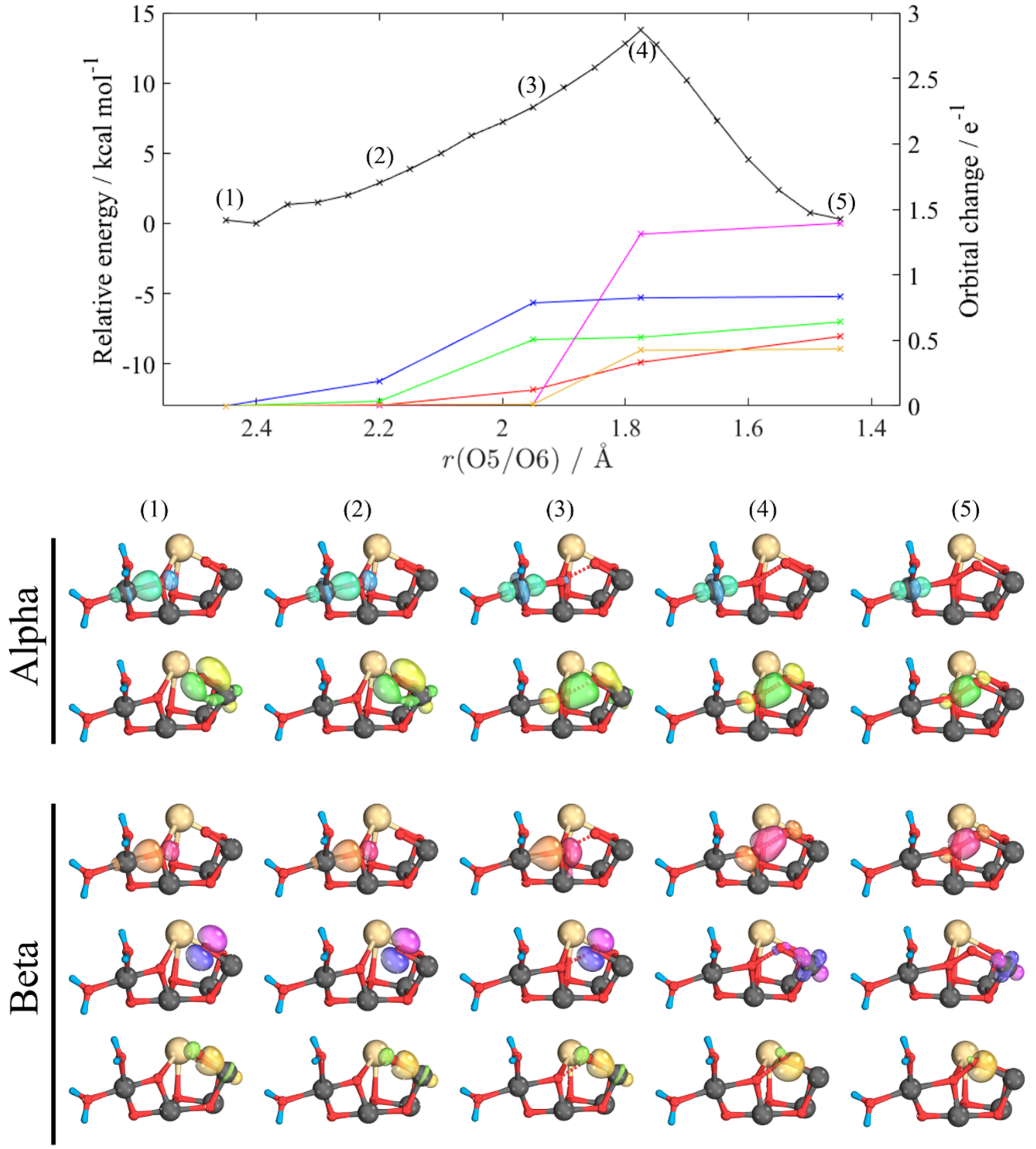

Figure S7: Intrinsic bond orbital (IBO) analysis of the $Ms = 3.5$ state of the oxo-oxo form in the $S_3Y_Z^{\bullet}$ state. Top, potential energy surface (PES) for O5-O6 bond formation (black) with corresponding IBO changes shown beneath, colour coded by orbital. IBOs are given at the labelled points on the PES showing α and β spin evolution.

Coordinates

Coordinates for HS $S_3Y_Z^{\bullet}$ oxo-hydroxo:

20	-33.03289	39.441702	363.612825
8	-33.154183	39.725494	366.054735
8	-31.776442	37.741259	364.838333
8	-33.745272	37.350394	366.3545
8	-32.289733	35.491427	363.279671
8	-33.875994	37.239564	363.340009
8	-35.175368	38.955538	364.59351
25	-34.800999	38.937531	366.35071
25	-32.098852	38.311057	366.517546
25	-32.996799	36.357407	364.664083
25	-33.544311	36.081546	362.011574
6	-32.875113	30.227234	362.898131
6	-33.417509	31.348585	362.00108
8	-32.577998	32.261697	361.668069
8	-34.613965	31.313638	361.622322
1	-33.689015	29.644162	363.33972
1	-32.241814	30.652549	363.685482
1	-32.248477	29.561478	362.289554
1	-29.571221	44.282104	356.71701
6	-30.377719	43.581095	356.44985
6	-31.228486	43.32719	357.653361
6	-30.75343	42.501515	358.715507

6	-32.505353	43.941195	357.788355
6	-31.47424	42.338335	359.874735
6	-33.247951	43.794591	358.936646
6	-32.742145	43.017004	360.047664
8	-33.389381	42.948895	361.142994
1	-30.952039	44.022334	355.629433
1	-29.889758	42.660385	356.105179
1	-29.791273	42.003797	358.602575
1	-32.888289	44.547037	356.968724
1	-31.105206	41.716283	360.68864
1	-34.220034	44.272675	359.043625
6	-30.294918	34.275884	358.026183
6	-31.26322	35.427561	358.133252
8	-32.492497	35.210195	358.223686
1	-30.65099	33.581714	357.257759
7	-30.738391	36.656574	358.158827
6	-31.498314	37.894333	358.274641
6	-31.171256	38.70269	359.529734
6	-31.746109	38.22168	360.859474
8	-31.655325	39.004661	361.826251
8	-32.288899	37.060827	360.885093
1	-29.737674	36.735463	358.059616
1	-32.56043	37.641673	358.244799
1	-30.084353	38.800695	359.661701

1	-31.556587	39.717346	359.393068
1	-30.288625	33.736038	358.98203
1	-29.275039	34.596055	357.791264
1	-31.270903	38.515955	357.398787
6	-36.337963	42.830694	366.005325
6	-36.307774	41.363032	365.69264
8	-36.885314	40.896099	364.686965
8	-35.641382	40.660247	366.548464
1	-35.33168	43.177789	366.262951
1	-36.977331	42.99713	366.881977
1	-36.746366	43.398268	365.164537
6	-36.143601	48.444125	363.03991
6	-35.60482	47.068168	362.872501
6	-35.358429	46.290409	361.768563
7	-35.192407	46.308222	363.954274
6	-34.708179	45.134747	363.545872
7	-34.806087	45.104739	362.219983
1	-36.444087	48.85357	362.071041
1	-35.38503	49.105474	363.477997
1	-35.530977	46.488111	360.718978
1	-34.260843	44.388913	364.193499
1	-34.452296	44.321649	361.652966
1	-37.018091	48.448279	363.702754
1	-35.233321	46.603519	364.919771

6	-39.840339	36.699968	365.631931
6	-38.593884	37.207145	366.253952
6	-37.333273	37.397515	365.747538
7	-38.538648	37.684918	367.55211
6	-37.304118	38.154181	367.803066
7	-36.552372	37.994371	366.720241
1	-39.624216	36.281611	364.643837
1	-40.575825	37.506323	365.509582
1	-36.940061	37.185172	364.76323
1	-36.9891	38.608832	368.731877
1	-40.306497	35.913785	366.239666
1	-39.304041	37.691811	368.210076
6	-33.839932	34.207894	371.940715
6	-34.126045	34.7448	370.572875
6	-33.47704	35.666432	369.783151
7	-35.22285	34.335637	369.827602
6	-35.248434	34.966237	368.649168
7	-34.19628	35.775448	368.603624
1	-34.711413	34.332829	372.595222
1	-32.999067	34.753665	372.379207
1	-32.573747	36.238979	369.967554
1	-35.985225	34.833643	367.867901
1	-33.988339	36.382909	367.785661
1	-33.579744	33.142328	371.903133

1	-35.911484	33.658616	370.12504
6	-33.607831	39.695786	370.356862
6	-33.565846	39.137262	368.965042
8	-32.44758	38.781308	368.48069
8	-34.657039	39.07399	368.321687
1	-34.61597	39.626602	370.772044
1	-33.312635	40.751472	370.284792
1	-32.88807	39.188187	371.005609
6	-29.063084	41.225436	366.547868
6	-30.139683	40.344015	365.929566
8	-30.630332	40.580518	364.813907
8	-30.507536	39.353143	366.695905
1	-28.52805	41.785543	365.775419
1	-28.367653	40.629007	367.146358
1	-29.575044	41.930685	367.216092
6	-30.495676	34.518567	367.650801
6	-31.259168	35.568989	366.898429
8	-31.162467	36.772329	367.308553
8	-31.97595	35.197897	365.92668
1	-29.9504	33.884383	366.942851
1	-31.209491	33.879223	368.1858
1	-29.804483	34.969367	368.366291
8	-30.576714	33.323705	363.252515
1	-31.201112	32.813817	362.692353

1	-31.089133	34.1409	363.412678
1	-32.844631	34.739869	359.888392
8	-30.700759	37.555831	370.313762
1	-30.091058	37.039248	369.770434
1	-31.176658	38.098214	369.657027
8	-34.925919	36.893133	361.050725
1	-34.999777	36.546163	360.140045
8	-33.19737	34.56715	360.787493
1	-32.989154	33.606867	361.11909
8	-36.964309	40.885241	361.913451
1	-37.045617	41.742092	361.473774
1	-37.078739	41.06559	362.869612
8	-38.620376	34.258136	359.811732
1	-38.407497	35.123538	359.409527
1	-37.752145	33.871569	360.034252
8	-35.938251	33.463129	360.476778
1	-35.709653	34.06732	361.206898
1	-35.440383	32.646146	360.738888
8	-32.584922	41.821985	363.451547
1	-31.625197	41.75853	363.618081
1	-32.733087	42.251866	362.572128
8	-35.286923	35.381578	358.582482
1	-35.392184	34.582115	359.148781
1	-34.365102	35.373069	358.269695

8	-37.126595	39.335731	359.19673
1	-36.171967	39.491735	359.034365
1	-37.268054	39.579158	360.124265
8	-32.457307	41.915669	367.732962
1	-32.686234	41.120826	367.215408
1	-32.604598	42.630575	367.086508
8	-34.580802	39.521957	361.627155
1	-35.428292	40.003865	361.752486
1	-34.815514	38.565804	361.443849
8	-34.32185	39.858466	358.919619
1	-34.237111	40.783754	358.655092
1	-34.249259	39.851204	359.902269
8	-32.855591	43.666998	365.543
1	-32.092445	44.252385	365.435917
1	-32.734561	42.972978	364.859402
6	-36.376179	33.719203	364.230246
6	-35.188022	34.616014	363.949845
8	-34.534548	35.105428	364.918054
8	-34.905567	34.827778	362.727356
1	-37.217102	34.02311	363.597823
1	-36.102454	32.692529	363.953848
1	-36.654508	33.761234	365.285534
8	-37.755414	36.660193	358.423575
1	-37.599575	37.562105	358.77242

1	-36.858486	36.276749	358.34706
1	-35.967731	39.532385	364.46498

Coordinates for HS $S_3Y_Z^{\bullet}$ peroxo:

20	-32.663575	39.669339	363.639969
8	-33.11521	39.726507	365.953209
8	-31.735823	37.752485	364.852515
8	-33.674834	37.29757	366.360416
8	-32.515144	35.712693	363.136868
8	-34.025668	37.574626	363.54914
8	-34.911824	38.595357	364.030786
25	-34.785573	38.920634	366.133914
25	-32.099077	38.325354	366.531221
25	-33.041363	36.445036	364.713794
25	-33.775394	36.124141	361.872696
6	-32.875113	30.227234	362.898131
6	-33.589375	31.312097	362.095935
8	-32.839793	32.122878	361.446978
8	-34.846258	31.343145	362.10505
1	-33.583349	29.603717	363.451962
1	-32.16986	30.696485	363.596048
1	-32.28838	29.597303	362.216731
1	-29.494876	44.250981	356.293472
6	-30.330363	43.552722	356.444251
6	-30.874809	43.675267	357.822522
6	-31.004896	42.533414	358.657786
6	-31.284282	44.948448	358.317583

6	-31.508889	42.640694	359.929588
6	-31.793909	45.084489	359.583411
6	-31.918979	43.929265	360.442338
8	-32.381136	44.03733	361.629269
1	-31.099111	43.837549	355.709617
1	-29.989041	42.537664	356.220885
1	-30.685875	41.566731	358.272555
1	-31.183684	45.819149	357.671102
1	-31.598885	41.776315	360.584561
1	-32.117461	46.050716	359.963973
6	-30.281131	34.310802	357.979278
6	-31.209911	35.496301	358.027059
8	-32.452415	35.317746	358.020143
1	-30.629829	33.613082	357.211232
7	-30.647775	36.705072	358.120441
6	-31.371752	37.966546	358.208408
6	-31.171256	38.70269	359.529734
6	-31.81327	38.14738	360.799812
8	-31.490781	38.685819	361.873865
8	-32.68089	37.213615	360.662612
1	-29.639969	36.750083	358.103234
1	-32.427661	37.761377	358.038889
1	-30.104253	38.849583	359.74031
1	-31.593712	39.710989	359.41717

1	-30.32985	33.792796	358.946221
1	-29.242578	34.593272	357.781267
1	-31.022484	38.615144	357.394922
6	-36.213704	42.923039	365.967991
6	-35.566068	41.65918	365.446187
8	-34.764344	41.719265	364.471007
8	-35.852452	40.58018	366.058844
1	-35.544881	43.348459	366.728405
1	-37.173565	42.705358	366.445069
1	-36.340866	43.661517	365.171112
6	-36.143601	48.444125	363.03991
6	-34.800659	47.826818	362.900852
6	-34.423245	46.520084	362.738288
7	-33.618149	48.552768	362.908161
6	-32.571444	47.736023	362.75898
7	-33.047759	46.500737	362.655365
1	-36.914724	47.676001	362.930509
1	-36.307557	49.208482	362.269778
1	-35.014557	45.616957	362.679335
1	-31.531489	48.032609	362.722881
1	-32.521609	45.647479	362.444325
1	-36.263983	48.924603	364.019712
1	-33.545214	49.554676	363.017992
6	-39.840339	36.699968	365.631931

6	-38.557867	37.183809	366.181732
6	-37.347759	37.437879	365.588267
7	-38.398714	37.546295	367.506368
6	-37.149625	38.007618	367.692439
7	-36.492874	37.952231	366.541283
1	-39.705677	36.358221	364.600539
1	-40.599712	37.493895	365.63352
1	-37.034052	37.323865	364.559325
1	-36.760338	38.392537	368.624342
1	-40.240708	35.860386	366.215183
1	-39.105526	37.481934	368.22358
6	-33.839932	34.207894	371.940715
6	-34.107752	34.744736	370.568453
6	-33.450606	35.670738	369.790816
7	-35.189597	34.33096	369.803329
6	-35.197017	34.964455	368.625334
7	-34.149605	35.780189	368.599957
1	-34.717955	34.337248	372.585626
1	-33.00181	34.750834	372.38789
1	-32.554487	36.249683	369.987877
1	-35.916959	34.827762	367.829156
1	-33.924678	36.38661	367.779505
1	-33.583312	33.141318	371.907932
1	-35.879633	33.649727	370.086957

6	-33.607831	39.695786	370.356862
6	-33.633393	39.235791	368.926457
8	-32.531109	38.767298	368.454339
8	-34.698984	39.316304	368.279705
1	-33.262907	38.883908	371.008385
1	-34.59688	40.033756	370.67471
1	-32.889057	40.518159	370.457907
6	-29.063084	41.225436	366.547868
6	-30.085386	40.29253	365.942121
8	-30.496142	40.455123	364.777187
8	-30.501727	39.364704	366.746338
1	-28.499686	41.748601	365.769706
1	-28.386352	40.68425	367.216668
1	-29.616144	41.962709	367.145214
6	-30.495677	34.518567	367.650801
6	-31.238702	35.573565	366.870109
8	-31.152087	36.766377	367.307886
8	-31.925854	35.216141	365.866333
1	-29.973497	33.841929	366.965616
1	-31.225404	33.923534	368.215166
1	-29.788305	34.975628	368.346115
8	-30.852805	33.50448	362.798545
1	-31.48117	32.925113	362.318058
1	-31.396658	34.294219	362.99878

1	-33.057459	34.634881	359.624133
8	-30.702198	37.574229	370.191983
1	-30.211784	37.050871	369.543612
1	-31.273434	38.124861	369.619288
8	-35.400765	36.850858	360.95555
1	-36.174208	36.220158	360.751
1	-35.374002	37.549535	360.262772
8	-33.529395	34.445656	360.459797
1	-33.243489	33.542668	360.821305
8	-34.940588	42.60203	361.859875
1	-34.130754	43.127285	361.745364
1	-35.01075	42.441464	362.828101
8	-37.355121	35.278006	360.33651
1	-37.394293	35.399443	359.359489
1	-36.962284	34.374447	360.450962
8	-35.925741	32.977009	360.254304
1	-35.1557	33.564317	360.134564
1	-35.606845	32.384471	360.987991
8	-31.8639	41.851111	363.088066
1	-31.023914	41.864494	363.577614
1	-31.963002	42.681431	362.579165
8	-34.588688	36.886999	357.39212
1	-34.467603	37.086399	356.452936
1	-33.818253	36.31205	357.631206

8	-36.480045	41.648199	359.259776
1	-36.12938	40.746873	359.146155
1	-36.100821	41.961873	360.0957
8	-32.6334	42.199484	367.160171
1	-32.799445	41.321013	366.757352
1	-32.792996	42.832908	366.440837
8	-34.239045	39.945423	361.653704
1	-34.533478	40.885441	361.646436
1	-34.898159	39.48144	362.209525
8	-35.218735	38.952239	359.200533
1	-34.784899	38.419989	358.501274
1	-34.543686	39.272395	359.829397
8	-33.30923	44.005781	364.834087
1	-32.460084	43.854806	364.39995
1	-33.829834	43.200166	364.613422
6	-36.376179	33.719203	364.230246
6	-35.265397	34.694342	363.94403
8	-34.572107	35.156637	364.903305
8	-35.095354	34.977542	362.721509
1	-37.249297	33.975254	363.621901
1	-36.027373	32.726332	363.914216
1	-36.636559	33.704886	365.291537
8	-37.162011	35.727544	357.59728
1	-37.754516	36.434479	357.307517

1	-36.259938	36.10755	357.512985

Coordinates for HS $S_3Y_Z^\bullet$ [O5O6]$^{3-}$:

20	-32.722931	39.566809	363.644467
8	-33.100415	39.735518	365.994491
8	-31.721248	37.728871	364.887766
8	-33.733716	37.368828	366.373027
8	-32.42026	35.638878	363.190039
8	-33.933781	37.507441	363.553716
8	-35.169605	38.940986	364.448427
25	-34.771971	38.971505	366.198231
25	-32.089983	38.310486	366.542914
25	-33.052706	36.440883	364.678269
25	-33.682922	36.070695	361.922832
6	-32.875113	30.227234	362.898131
6	-33.556413	31.299801	362.06122
8	-32.779616	32.104335	361.438756
8	-34.812512	31.322491	362.017522
1	-33.60191	29.615595	363.441326
1	-32.184797	30.700046	363.608433
1	-32.274861	29.582396	362.242654
1	-29.480452	44.241064	356.329824
6	-30.330363	43.552722	356.444251
6	-30.903698	43.653552	357.812333
6	-31.032025	42.503249	358.636692
6	-31.341066	44.916339	358.309495

6	-31.561291	42.593649	359.899608
6	-31.876363	45.035204	359.566287
6	-31.999493	43.871504	360.414715
8	-32.482809	43.964347	361.593856
1	-31.076471	43.865958	355.698296
1	-29.997003	42.538293	356.206281
1	-30.69225	41.544004	358.250936
1	-31.241807	45.793561	357.671763
1	-31.649789	41.724668	360.548722
1	-32.222636	45.993979	359.945638
6	-30.268518	34.319778	357.994902
6	-31.209911	35.496301	358.027059
8	-32.449694	35.30513	357.996873
1	-30.595638	33.620513	357.218861
7	-30.661168	36.709335	358.126131
6	-31.402929	37.958138	358.220169
6	-31.171256	38.70269	359.529734
6	-31.738669	38.125893	360.825437
8	-31.384506	38.661207	361.895214
8	-32.583881	37.173757	360.702455
1	-29.653777	36.765538	358.130137
1	-32.459394	37.730093	358.085492
1	-30.101399	38.888719	359.690784
1	-31.63172	39.696118	359.434041

1	-30.32899	33.798243	358.959229
1	-29.229453	34.613582	357.816796
1	-31.09111	38.606642	357.391163
6	-36.375773	42.879706	365.824565
6	-35.566068	41.65918	365.446187
8	-34.776267	41.698585	364.475831
8	-35.728561	40.643313	366.222251
1	-35.748048	43.509302	366.469677
1	-37.273689	42.604675	366.384126
1	-36.640131	43.458118	364.934205
6	-36.143601	48.444125	363.03991
6	-34.814686	47.802992	362.881597
6	-34.465492	46.488965	362.717188
7	-33.618809	48.506059	362.868453
6	-32.590566	47.668138	362.705908
7	-33.092557	46.442299	362.613248
1	-36.929265	47.687224	362.959031
1	-36.312857	49.200832	362.263363
1	-35.074409	45.597034	362.669901
1	-31.545894	47.944841	362.652072
1	-32.589725	45.575294	362.398318
1	-36.234821	48.939404	364.015414
1	-33.524757	49.50677	362.97249
6	-39.840339	36.699968	365.631931

6	-38.575367	37.219323	366.214487
6	-37.341705	37.458064	365.660968
7	-38.468141	37.647651	367.527526
6	-37.22908	38.129428	367.739633
7	-36.525687	38.028257	366.619427
1	-39.661533	36.326202	364.61863
1	-40.604274	37.487346	365.578957
1	-36.984642	37.29881	364.653289
1	-36.881277	38.556963	368.669695
1	-40.252629	35.876512	366.229335
1	-39.202173	37.616037	368.219187
6	-33.839932	34.207894	371.940715
6	-34.124598	34.760386	370.577323
6	-33.468468	35.681333	369.79244
7	-35.228571	34.370467	369.831724
6	-35.250992	35.012624	368.658836
7	-34.190757	35.811192	368.616984
1	-34.706344	34.34115	372.600367
1	-32.988506	34.737279	372.379057
1	-32.557498	36.241083	369.974615
1	-35.990703	34.89508	367.877678
1	-33.97765	36.421977	367.799581
1	-33.596463	33.138764	371.893476
1	-35.923182	33.697833	370.124955

6	-33.607831	39.695786	370.356862
6	-33.596437	39.222637	368.92243
8	-32.488772	38.776279	368.479258
8	-34.655879	39.276969	368.23543
1	-33.26049	38.889552	371.01319
1	-34.605933	40.022023	370.658698
1	-32.901586	40.528687	370.454189
6	-29.063084	41.225436	366.547868
6	-30.090739	40.287984	365.948494
8	-30.520678	40.451989	364.792484
8	-30.491261	39.35098	366.755397
1	-28.511334	41.752629	365.764162
1	-28.376446	40.683761	367.206117
1	-29.611095	41.95888	367.154396
6	-30.495677	34.518567	367.650801
6	-31.272931	35.561997	366.895347
8	-31.185957	36.758062	367.340416
8	-31.980878	35.206528	365.91526
1	-29.991092	33.846868	366.947841
1	-31.199169	33.915328	368.239588
1	-29.767499	34.978034	368.322961
8	-30.826246	33.386615	362.948589
1	-31.441815	32.850652	362.40478
1	-31.353705	34.18947	363.140196

1	-32.984578	34.611884	359.603798
8	-30.636489	37.528496	370.21292
1	-30.198656	36.995662	369.534966
1	-31.202492	38.112895	369.673031
8	-35.315018	36.744732	360.982315
1	-36.070209	36.096659	360.775484
1	-35.289262	37.428975	360.272267
8	-33.442356	34.413759	360.443717
1	-33.15808	33.508457	360.797977
8	-34.989792	42.428822	361.832229
1	-34.200837	42.980885	361.698008
1	-35.053916	42.30753	362.806565
8	-37.264631	35.151412	360.356367
1	-37.353318	35.298519	359.386672
1	-36.841464	34.253928	360.412755
8	-35.793333	32.902538	360.104072
1	-35.033783	33.512347	360.032193
1	-35.499967	32.318907	360.858378
8	-31.938088	41.771326	363.065194
1	-31.099301	41.789063	363.557264
1	-32.046829	42.606278	362.568099
8	-34.636484	36.770111	357.32056
1	-34.525234	36.97289	356.381031
1	-33.84107	36.232123	357.564505

8	-36.498974	41.432834	359.275959
1	-36.126453	40.53908	359.171926
1	-36.123053	41.767464	360.105439
8	-32.661882	42.206775	367.199037
1	-32.798068	41.329153	366.784472
1	-32.798392	42.841288	366.475998
8	-34.227937	39.789063	361.661038
1	-34.566457	40.714627	361.647373
1	-34.939791	39.259179	362.057882
8	-35.17546	38.76007	359.197876
1	-34.823406	38.24484	358.439066
1	-34.429336	39.099512	359.722586
8	-33.301046	43.998029	364.846669
1	-32.435868	43.845326	364.44578
1	-33.815429	43.198637	364.603818
6	-36.376179	33.719203	364.230246
6	-35.22546	34.641478	363.937464
8	-34.567746	35.154306	364.898373
8	-34.988666	34.835165	362.712556
1	-37.232935	34.010327	363.613544
1	-36.077042	32.70729	363.926009
1	-36.64742	33.730238	365.288988
8	-37.215077	35.621458	357.609378
1	-37.806567	36.340547	357.348919

1	-36.311424	35.986584	357.490446

References

(1) Rummel, F.; O'Malley, P. J. How Nature Makes O2: an Electronic Level Mechanism for Water Oxidation in Photosynthesis. *The Journal of Physical Chemistry B* **2022**, *126*, 8214–8221.
